# Evidence That Bank Vole PrP Is a Universal Acceptor for Prions

**DOI:** 10.1371/journal.ppat.1003990

**Published:** 2014-04-03

**Authors:** Joel C. Watts, Kurt Giles, Smita Patel, Abby Oehler, Stephen J. DeArmond, Stanley B. Prusiner

**Affiliations:** 1 Institute for Neurodegenerative Diseases, University of California, San Francisco, San Francisco, California, United States of America; 2 Department of Neurology, University of California, San Francisco, San Francisco, California, United States of America; 3 Department of Pathology, University of California, San Francisco, San Francisco, California, United States of America; University of Edinburgh, United Kingdom

## Abstract

Bank voles are uniquely susceptible to a wide range of prion strains isolated from many different species. To determine if this enhanced susceptibility to interspecies prion transmission is encoded within the sequence of the bank vole prion protein (BVPrP), we inoculated Tg(M109) and Tg(I109) mice, which express BVPrP containing either methionine or isoleucine at polymorphic codon 109, with 16 prion isolates from 8 different species: humans, cattle, elk, sheep, guinea pigs, hamsters, mice, and meadow voles. Efficient disease transmission was observed in both Tg(M109) and Tg(I109) mice. For instance, inoculation of the most common human prion strain, sporadic Creutzfeldt-Jakob disease (sCJD) subtype MM1, into Tg(M109) mice gave incubation periods of ∼200 days that were shortened slightly on second passage. Chronic wasting disease prions exhibited an incubation time of ∼250 days, which shortened to ∼150 days upon second passage in Tg(M109) mice. Unexpectedly, bovine spongiform encephalopathy and variant CJD prions caused rapid neurological dysfunction in Tg(M109) mice upon second passage, with incubation periods of 64 and 40 days, respectively. Despite the rapid incubation periods, other strain-specified properties of many prion isolates—including the size of proteinase K–resistant PrP^Sc^, the pattern of cerebral PrP^Sc^ deposition, and the conformational stability—were remarkably conserved upon serial passage in Tg(M109) mice. Our results demonstrate that expression of BVPrP is sufficient to engender enhanced susceptibility to a diverse range of prion isolates, suggesting that BVPrP may be a universal acceptor for prions.

## Author Summary

Prions are infectious proteins that cause devastating neurodegenerative diseases in both humans and animals. Unlike other rodents, bank voles are highly susceptible to prions from many different species, suggesting that bank voles do not impose a “species barrier,” which normally restricts the transmission of prions from one species to another. We were curious as to whether the unprecedented promiscuity of bank voles for prions is due to the specific prion protein sequence expressed, or to some other factor inherent to bank vole physiology. To answer this question, we inoculated transgenic mice that express bank vole prion protein [Tg(BVPrP) mice] with a diverse set of prions deriving from eight different species. Like bank voles, Tg(BVPrP) mice were highly susceptible to prions from all species tested, demonstrating that the BVPrP sequence mediates the enhanced susceptibility of bank voles to prions. Because the amino acid sequences of mouse and BVPrP differ at only eight positions, our results demonstrate that alterations to a small subset of residues within PrP can have a profound effect on the susceptibility of an organism to prions from another species.

## Introduction

Prions, or proteinaceous infectious particles, are self-propagating protein conformations that cause a variety of fatal neurodegenerative illnesses. Prions composed of the prion protein (PrP) cause Creutzfeldt-Jakob disease (CJD) in humans, scrapie in sheep, chronic wasting disease (CWD) in cervids, and bovine spongiform encephalopathy (BSE) [1], [2], [3]. In these diseases, cellular PrP (PrP^C^), which is a glycosylphosphatidylinositol (GPI)-anchored membrane protein, undergoes a conformational conversion into a β-sheet-rich, aggregation-prone isoform, termed PrP^Sc^ [4], [5]. Accumulation of PrP^Sc^ within the central nervous system (CNS) results in profound neurological dysfunction as well as neuropathological changes, which include spongiform (vacuolar) degeneration, astrocytic gliosis, and neuronal loss. In contrast to PrP^C^, which is sensitive to protease digestion, the most commonly encountered forms of PrP^Sc^ are partially resistant to digestion with proteases, producing a truncated fragment referred to as PrP 27–30 [6]. Distinct strains of prions can be distinguished and classified by the incubation periods upon inoculation of laboratory animals, differences in neuroanatomic target areas and patterns of PrP^Sc^ deposition within the brain, and biochemical properties, including the molecular weight of PrP 27–30 [7], [8]. It is believed that the properties of distinct prion strains are enciphered within the conformation of PrP^Sc^ [9], [10]. In some instances, it is more appropriate to refer to prion strains as “isolates” if they have not been serially passaged.

The intraspecies transmission of various prion strains or isolates is generally an efficient process, in which 100% of the inoculated animals develop CNS disease, the incubation period is relatively uniform, neuropathologic patterns are similar, and biochemical properties of PrP^Sc^ are maintained. In contrast, the interspecies transmission of prions is usually an inefficient process, in which only a fraction of inoculated animals develop signs of neurologic dysfunction, resulting in more variable and prolonged incubation periods [11], [12]. Furthermore, the properties of prion strains or isolates are frequently altered upon initial passage in a different species [13]. Upon second passage, the incubation periods are shorter, and the biochemical and neuropathological properties of the prion isolate are stabilized. This phenomenon is what is referred to as the “species barrier” for prion replication [14]. At the molecular level, the species barrier was initially believed to be governed entirely by the sequence of PrP, with interspecies differences in the amino acid sequence of PrP hindering disease transmission [15]. For instance, transgenic (Tg) mice expressing hamster or human PrP are susceptible to hamster or human prions, respectively, whereas wild-type (wt) mice are largely resistant to prions from either species [16], [17].

With further study, it became clear that the initially monastic view of the species barrier was incomplete: in particular, differences in the sequences of PrP^Sc^ in the inoculum and PrP^C^ in the host were insufficient to explain all aspects of prion transmission from one host to another. For example, the MM1 subtype of sporadic (s) CJD prions transmitted to Tg mice expressing the M129 variant of human PrP in ∼200 days while variant (v) CJD prions required more than 600 days [18]. Conversely, Tg mice expressing bovine PrP exhibited signs of neurological dysfunction at ∼270 days after inoculation with vCJD prions, but remained well for greater than 500 days after inoculation with sCJD(MM1) prions [19], [20], [21]. Importantly, the proteins comprising vCJD and sCJD(MM1) prions had the same amino acid sequence, arguing that an additional “barrier” must be invoked to explain the differences in transmission efficiency described above, which might best be called a “strain barrier” to reflect distinct conformations of PrP^Sc^ molecules. Together, the species and strain barriers have been called “transmission barriers,” where a given PrP^C^ sequence is capable of propagating only a distinct subset of PrP^Sc^ conformations [20], [22]. When the PrP^C^ and PrP^Sc^ conformations are compatible, efficient disease transmission occurs.

Unlike other rodents, bank voles (*Myodes glareolus*) are susceptible to prions from a diverse range of species, including humans [23], [24], [25], [26], [27]. This suggests that species and possibly strain barriers are greatly attenuated in bank voles, an observation that has been recapitulated *in vitro* using protein misfolding cyclic amplification (PMCA) [28]. Two explanations seem plausible for the promiscuity of bank voles for replicating prions originating in diverse species: first, the presence of an especially permissive prion replication cofactor [17], [29], [30], [31], [32] and second, a broadly compatible bank vole PrP (BVPrP) sequence. The latter would seem to be the more parsimonious explanation where the amino acid sequence of BVPrP facilitates adoption of self-propagating conformations both spontaneously and upon exposure to exogenous prions [24], [33]. The sequence of the mature processed form of BVPrP, in which the N- and C-terminal signal peptides have been removed, differs from that of mouse PrP at only eight positions [25]. Notably, the high-resolution structure of bank vole PrP^C^ revealed the presence of a “rigid loop” but no remarkable characteristics that might confer its unique replication behavior [34].

To determine the range of prion susceptibility conferred by BVPrP expression, we challenged Tg mice expressing BVPrP with 16 prion isolates from 8 different species. BVPrP is polymorphic at codon 109, where either a methionine (M) or isoleucine (I) residue can be present [35]. Tg(BVPrP) mice expressing either the M109 or I109 polymorphic variant of BVPrP were susceptible to a wide range of prion isolates derived from many species, confirming that the enhanced susceptibility of bank voles to prions with different PrP sequences is mediated by the sequence of BVPrP.

## Results

### Transmission of prions to Tg(M109) mice

Because Tg mice that express the I109 allotype of BVPrP develop age-dependent signs of spontaneous neurologic illness [33], we initially focused our studies on Tg mice expressing the M109 allotype. Tg(BVPrP,M109)22019 mice, denoted Tg(M109) mice, express BVPrP at ∼5 times the level of PrP expression found in wt mice and did not develop any signs of spontaneous neurologic illness up to 500 days of age [33]. We inoculated the Tg(M109) mice with 16 different prion isolates derived from humans, cattle, elk, sheep, guinea pigs, hamsters, mice or meadow voles (MV) (**Table 1**). The following prion isolates were tested: sCJD (three subtypes: MM1, MM2, and VV2); vCJD; sCJD(MM1) prions passaged in Tg mice expressing the M129 variant of human PrP [Tg(HuPrP) mice]; cattle BSE; elk CWD; sheep scrapie isolate SSBP/1; sCJD(MM1) prions passaged in guinea pigs; hamster-adapted scrapie strain Sc237; mouse-adapted scrapie strain RML; mouse-adapted BSE strain 301V [maintained in mice expressing either the PrP-A or PrP-B allotype of mouse PrP and denoted 301V(A) and 301V(B), respectively]; MV-adapted RML; and MV-adapted Sc237 prions. Remarkably, 119 of 120 inoculated Tg(M109) mice developed signs of neurologic dysfunction consistent with prion disease, with mean incubation periods ranging from 50 days to just under 400 days (**Table 1**). The relatively short incubation periods and high transmission efficiencies for this diverse set of prion isolates in Tg(M109) mice suggest that these mice, like bank voles, do not impose a barrier for interspecies prion transmission. To determine the reproducibility of these findings, we utilized another Tg line denoted Tg(BVPrP,M109)3118 mice, which express BVPrP at ∼2.5 times the level of PrP expression in wt mice. Like the Tg(M109)22019 mice, the Tg(M109)3118 mice were also susceptible to MV-, mouse-, hamster-, and human-derived prion isolates (**Table S1**). Tg(MoPrP) mice, which overexpress mouse PrP at ∼4–5 times the level in wt mice, did not exhibit a general susceptibility to prions (**Table S2**), arguing that the increased susceptibility of Tg(M109) mice to diverse prion isolates cannot be attributed to PrP overexpression.

**Table 1:** Transmission of diverse prion isolates to Tg(BVPrP,M109)22019 mice.*

Prion isolate	PrP^Sc^ sequence	1^st^ passage		2^nd^ passage	
		Mean incubation period ± SEM (d)	Signs of neurologic dysfunction (*n*/*n*_0_)	Mean incubation period ± SEM (d)	Signs of neurologic dysfunction (*n*/*n*_0_)
RML → MV	meadow vole	50±3	8/8	*nd*	*nd*
RML	mouse (PrP-A)	73±1	8/8	52±1	8/8
301V(A)	mouse (PrP-A)	124±4	8/8	79±1	8/8
301V(B)	mouse (PrP-B)	138±5	7/7	78±1	8/8
Sc237 → MV	meadow vole	82±3	8/8	*nd*	*nd*
Sc237	hamster	96±5	7/7	77±4	8/8
Scrapie SSBP/1	sheep	383±26	8/8	82±3	7/7
CWD	elk	249±25	7/8†	146±5	8/8
BSE	cattle	368±10	7/7	64±2	8/8
vCJD	human	330±15	7/7	40±2	7/7
sCJD(MM1) case i	human	196±4	8/8	175±12	8/8
sCJD(MM1) case ii	human	204±2	7/7	*nd*	*nd*
sCJD(MM1) → Tg(HuPrP)	human	240±4	7/7	*nd*	*nd*
sCJD(MM1) → GP	guinea pig	193±15	8/8	*nd*	*nd*
sCJD(MM2)	human	244±30	7/7	89±4	8/8
sCJD(VV2)	human	394±18	7/7	103±4	7/7

*n, *number of positive mice;* n*_0_, number of inoculated mice; nd, not determined.*

† *One mouse remained free of clinical signs at 503 days postinoculation.*

In the Tg(M109) mice, two cases of sCJD(MM1) prions produced incubation times of ∼200 days; on second passage, the incubation period decreased modestly to 175 days. Relatively larger reductions in the incubation times on second passage were observed with other prion isolates. For example, sCJD(MM2) prions gave an incubation time of ∼240 days on first passage, which decreased to 90 days on second passage, and sCJD(VV2) prions decreased from ∼400 days on first passage to ∼100 days on second passage. Most interesting among the human isolates was vCJD, with an initial incubation time of 330 days, which decreased to 40 days on second passage in Tg(M109) mice, an 8-fold reduction upon serial transmission. Notably, BSE prions, from which vCJD prions are derived, exhibited an incubation time of ∼370 days on first passage and decreased to ∼65 days on second passage. CWD prions from elk produced neurological dysfunction in ∼250 days on first passage and ∼150 days on second passage in Tg(M109) mice. Sc237 prions from Syrian hamsters inoculated into Tg(M109) mice produced an incubation time of ∼95 days on first passage, which decreased to ∼75 days on second passage. When RML prions from wt mice were inoculated into Tg(M109) mice, the incubation time was ∼75 days but decreased to ∼50 days on second passage. These results suggest that transmission barriers still exist for some strains despite a general susceptibility of Tg(M109) mice to many different prion isolates.

**Figure 1:**
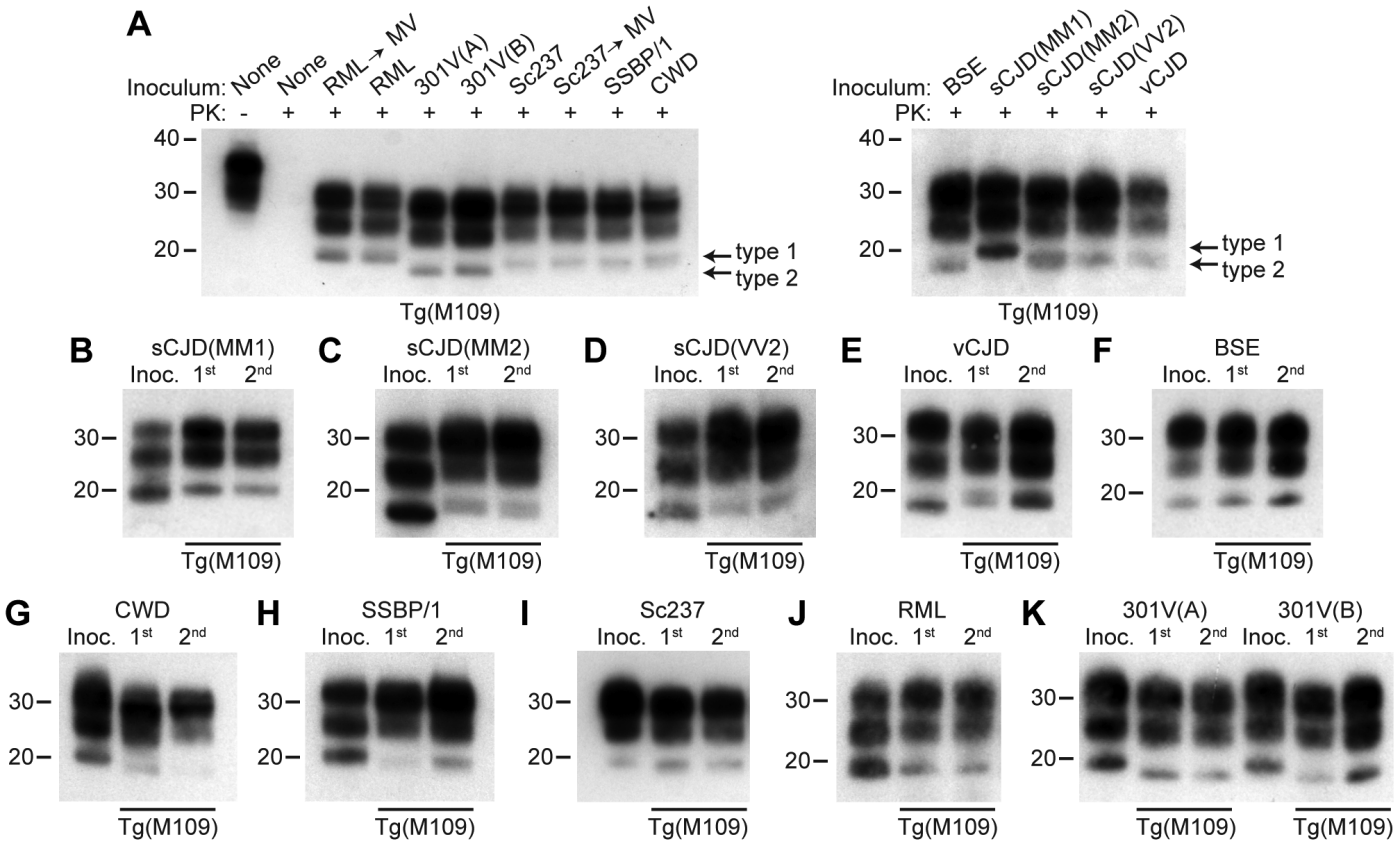
Analysis of PK-resistant PrP^Sc^ in Tg(M109) mice inoculated with diverse prion isolates. Except for one undigested lane (−), brain samples were digested with PK (+ in panel A). All inoculation experiments resulted in the generation of PK-resistant PrP^Sc^. (**A**) Detergent-extracted brain homogenates from Tg(M109) mice that were inoculated with the indicated prion isolates. Brain homogenate from an uninoculated, 97-d-old mouse (None) is shown as a control. (**B**–**K**) PK-resistant PrP^Sc^ in the inocula (Inoc.), and after 1^st^ and 2^nd^ passages in Tg(M109) mice. Inocula were sCJD(MM1) (**B**); sCJD(MM2) (**C**); sCJD(VV2) (**D**); vCJD (**E**); BSE (**F**); CWD (**G**); scrapie SSBP/1 (**H**); Sc237 (**I**); RML (**J**); 301V(A) or 301V(B) (**K**) prions. With the exception of 301V, the electrophoretic mobility of PK-resistant PrP^Sc^ for each strain following passage in Tg(M109) mice was similar to the original isolate. In all panels, loading quantities were adjusted prior to immunoblotting to give similar signal intensities across all samples. Molecular weight measurements are shown in kDa. PrP was detected using the antibody HuM-P.

Proteinase K (PK)-resistant PrP^Sc^ was found in the brains of all clinically ill Tg(M109) mice inoculated with each of the prion isolates tested (**Figure 1A**). Furthermore, spongiform degeneration and prominent astrocytic gliosis were found in the brains of Tg(M109) mice inoculated with each of the different isolates (**Figure S1**), confirming that these mice developed prion disease. However, levels of PK-resistant PrP^Sc^ were much lower in RML-inoculated Tg(M109) mice than in RML-inoculated wt mice or in RML-infected Tg(MoPrP) mice (**Figure S2A**). Similarly, levels of PK-resistant PrP^Sc^ following challenge of Tg(M109) mice with Sc237 prions were much lower than in Sc237-infected hamsters or in Sc237-inoculated Tg mice overexpressing hamster PrP [Tg(SHaPrP) mice] (**Figure S2B**). Levels of PK-resistant PrP^Sc^ remained low upon second passage of RML or Sc237 prions in Tg(M109) mice. However, this was not true for all the prion isolates analyzed: substantially higher levels of PK-resistant PrP^Sc^ were observed in the brains of BSE- and vCJD-inoculated Tg(M109) mice (**Figure S2C**).

### Strain-specified molecular weights of protease-resistant PrP^Sc^

Prion strains can be classified according to the electrophoretic mobility of the unglycosylated band of PK-resistant PrP^Sc^, migrating to either ∼21 kDa or ∼19 kDa, respectively termed type 1 and type 2 strains, similar to the nomenclature for sCJD prions [8]. In Tg(M109) mice, type 1 strains migrated to ∼20 kDa and type 2 strains migrated to ∼19 kDa (**Figure 1A**). In general, the electrophoretic mobilities observed for the original prion isolates were conserved upon transmission to Tg(M109) mice (**Figure 1B–K**). For instance, RML, Sc237, CWD, scrapie SSBP/1, and sCJD(MM1) are type 1 strains and exhibited a type 1 pattern upon transmission to Tg(M109) mice. Similarly, BSE, sCJD(MM2), sCJD(VV2), and vCJD are type 2 strains and generated type 2 strains following transmission to Tg(M109) mice. Slight alterations in the size of PK-resistant PrP^Sc^ were observed for the sCJD(MM1), CWD, and SSBP/1 isolates upon propagation in Tg(M109) mice (**Figure 1B, G–H**), and type 2 PrP^Sc^ in Tg(M109) mice had a slightly larger molecular mass compared to the type 2 PrP^Sc^ in the original human inocula (**Figure 1C–F**). However, of the 11 isolates analyzed, only 2 clearly changed strain type upon passage in Tg(M109) mice: both the 301V(A) and 301V(B) isolates exhibited a type 2 pattern in Tg(M109) mice whereas the original isolates were type 1 strains (**Figure 1K**). Thus, Tg(M109)-passaged 301V prions more closely resembled the BSE isolate from which the 301V strain was originally derived.

Another method for discriminating prion strains is the comparison of the relative abundances of di-, mono-, and unglycosylated PK-resistant PrP^Sc^. The most abundant glycoform for all prion isolates was diglycosylated PrP^Sc^ following passage in Tg(M109) mice (**Figure 1A**). For prion isolates with high levels of diglycosylated PrP^Sc^ (such as Sc237, 301V, CWD, BSE, and vCJD), the glycoform ratios appeared to be conserved upon serial passage in Tg(M109) mice (**Figure 1E–G, I, K**). In contrast, for prion isolates that did not exhibit high levels of diglycosylated PrP^Sc^, such as sCJD(MM1), sCJD(MM2), sCJD(VV2), SSBP/1, and RML, the relative abundance of diglycosylated PrP^Sc^ increased upon propagation in Tg(M109) mice (**Figure 1B–D, H, J**).

### Strain-specific neuropathological patterns of PrP^Sc^ deposition

**Figure 2:**
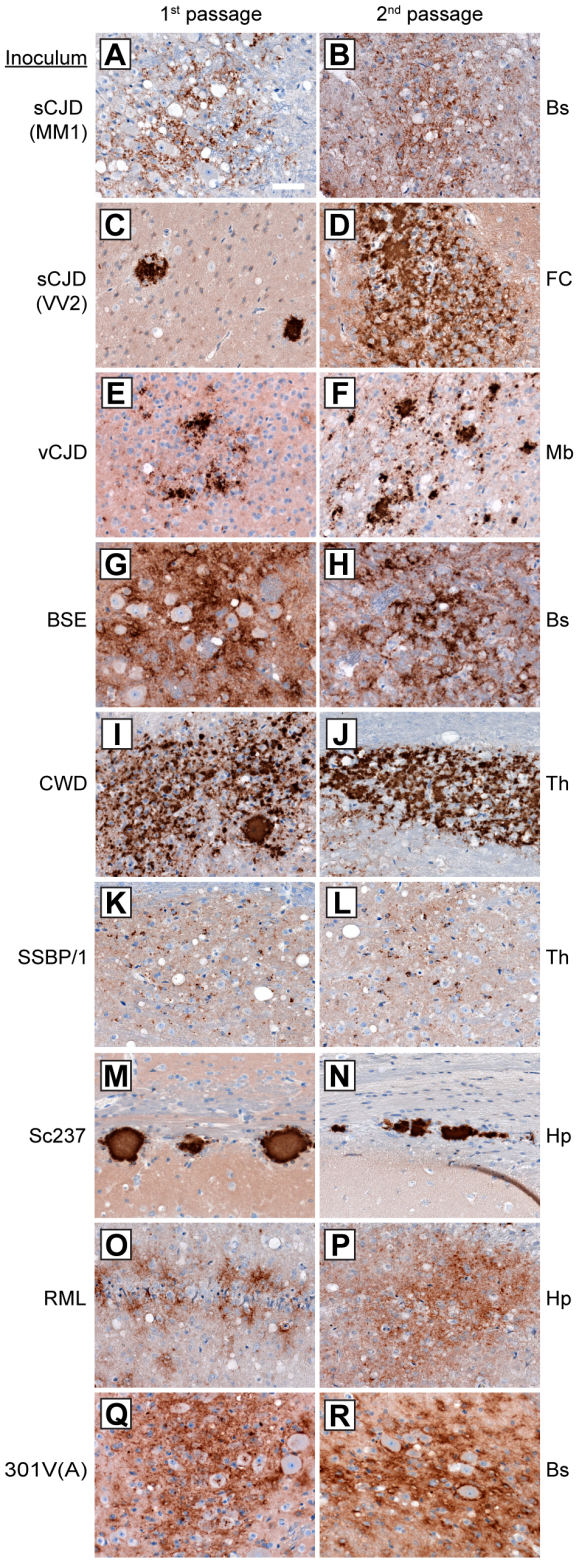
Patterns of cerebral PrP^Sc^ deposition in Tg(M109) mice inoculated with diverse prion isolates. Patterns of PrP^Sc^ deposition in the brain following first (left column) and second (right column) passage of sCJD(MM1) (**A**, **B**); sCJD(VV2) (**C**, **D**); vCJD (**E**, **F**); BSE (**G**, **H**); CWD (**I**, **J**); scrapie SSBP/1 (**K**, **L**); Sc237 (**M**, **N**); RML (**O**, **P**); or 301V(A) (**Q**, **R**) prions in Tg(M109) mice. For individual prion isolates, patterns of PrP^Sc^ deposition were maintained upon serial passage in Tg(M109) mice and were generally reminiscent of the PrP^Sc^ deposition characteristics of the original isolates. Brain regions with characteristic PrP^Sc^ deposition for the inoculum are shown: Bs, brainstem; FC, frontal cortex; Mb, midbrain; Th, thalamus; Hp, hippocampus. PrP^Sc^ deposits were detected using the antibody HuM-D18. Scale bar in *A* represents 50 µm and applies to all panels.

To further investigate whether the properties of the prion isolates were conserved upon transmission in Tg(M109) mice, we examined the patterns of PrP^Sc^ deposition in the brains of prion-inoculated Tg(M109) mice. PrP^Sc^ deposition was found in the brains of all clinically ill Tg(M109) mice inoculated with each of the prion isolates tested (**Figure 2A–R**), although the level of PrP^Sc^ deposition in Tg(M109) mice was typically less than what is observed in other experimentally inoculated laboratory animals. Generally, the characteristic pattern of PrP^Sc^ deposition for a given prion isolate was conserved following one or two passages in Tg(M109) mice.

For the human inocula, the “synaptic” pattern of PrP^Sc^ deposition observed with the sCJD(MM1) subtype and the plaque-like deposition of PrP^Sc^ commonly observed with sCJD(VV2) were both recapitulated in Tg(M109) mice (**Figure 2A, C**). PrP^Sc^ plaques were observed in the vicinity of vacuolation in vCJD-inoculated Tg(M109) mice (**Figure 2E**), which is somewhat reminiscent of the “florid” PrP^Sc^ plaques present in the brains of vCJD patients [36]. Notably, the presence of florid plaques in vCJD-inoculated animals is species-dependent and their absence does not necessarily imply lack of strain fidelity [37], [38].

The neuropathological signature of RML prions in mice is the diffuse deposition of PrP^Sc^ in the hippocampus; this pattern of PrP^Sc^ deposition was also observed in RML-inoculated Tg(M109) mice (**Figure 2O**). Similarly, plaque-like PrP^Sc^ aggregates in the corpus callosum, which is the hallmark of the Sc237 strain, were observed in Sc237-inoculated Tg(M109) mice (**Figure 2M**), and the thalamic plaque-like PrP^Sc^ deposits in CWD-inoculated Tg(M109) mice (**Figure 2I**) resembled those present in CWD-inoculated Tg mice expressing elk PrP [39]. We conclude that for the majority of prion isolates, the neuropathological signatures of PrP^Sc^ deposition were maintained upon transmission to Tg(M109) mice. Additionally, the pattern of cerebral PrP^Sc^ deposition on first passage was indistinguishable for each isolate when compared to the second passage in Tg(M109) mice (**Figure 2**, compare left and right columns).

### Conformational stabilities of prion strains

**Figure 3:**
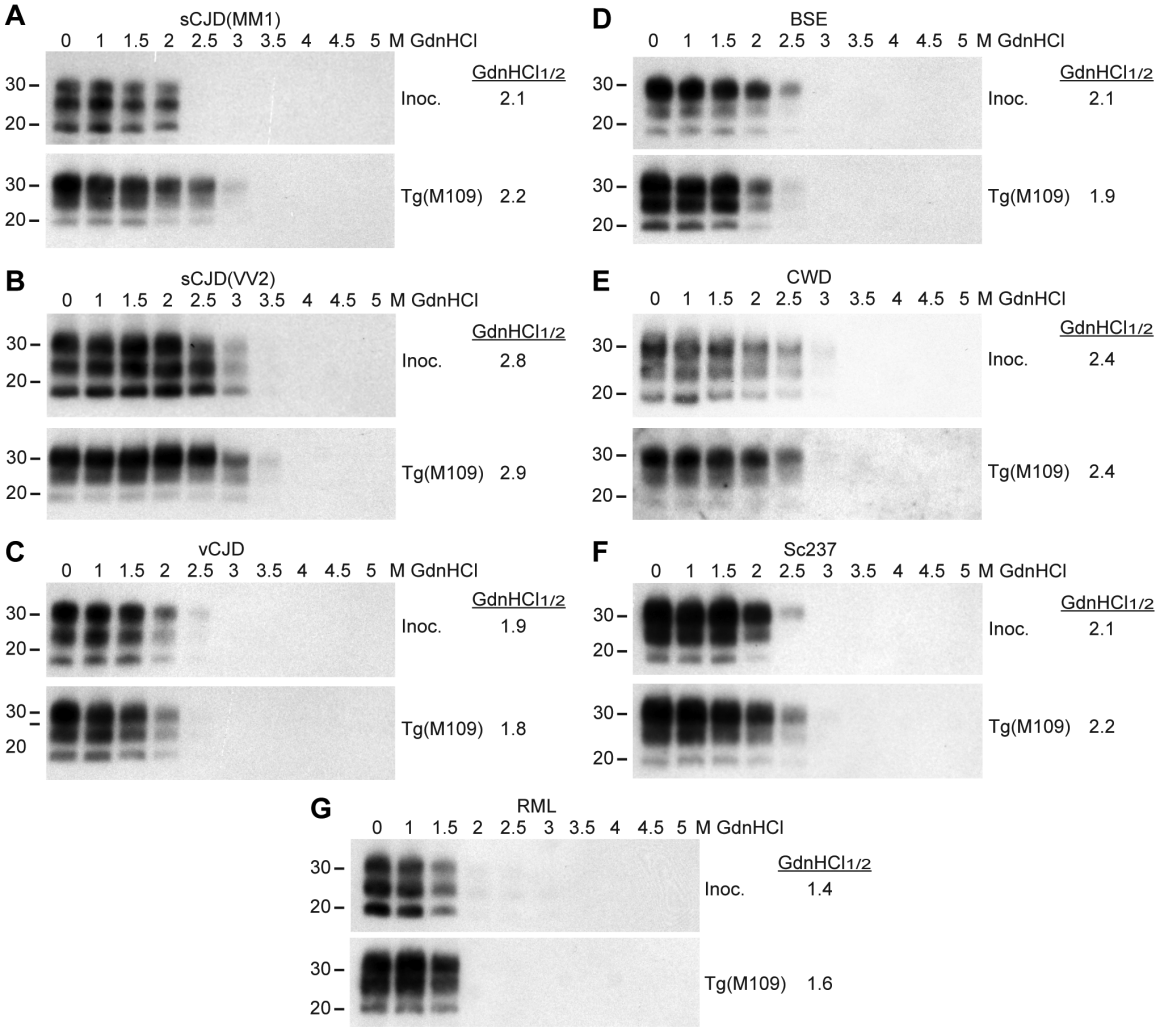
Conformational stability measurements of Tg(M109)-passaged prion isolates. Conformational stability assays were performed on the original prion isolates used as inocula (Inoc.) and on brain homogenates from clinically ill Tg(M109) mice following two passages of sCJD(MM1) (**A**), sCJD(VV2) (**B**), vCJD (**C**), BSE (**D**), CWD (**E**), Sc237 (**F**), or RML (**G**) prions. The calculated GdnHCl_1/2_ values for each isolate remained similar. For all panels, molecular weight measurements are shown in kDa. PK-resistant PrP^Sc^ was detected using the antibody HuM-P.

The conformational stability of PrP^Sc^ molecules, which is a measure of their ability to resist denaturation by guanidine hydrochloride (GdnHCl) [40], was used to characterize the prion strains transmitted to Tg(M109) mice. We performed conformational stability assays on the original inocula and after serial transmission through Tg(M109) mice by titrating the stability of protease-resistant PrP^Sc^ using GdnHCl denaturation (**Figure 3**). Before and after two passages in Tg(M109) mice, sCJD(MM1), vCJD, BSE, and Sc237 prions exhibited GdnHCl_1/2_ values of ∼2 M (**Figure 3A, C, D, F**) while sCJD(VV2) prions had GdnHCl_1/2_ values of ∼2.8 M (**Figure 3B**). CWD prions, either before or after passaging in Tg(M109) mice, were intermediate with GdnHCl_1/2_ values of 2.4 M (**Figure 3E**), whereas RML prions exhibited the lowest conformational stability of ∼1.5 M, which was unchanged upon propagation in Tg(M109) mice (**Figure 3G**). These findings argue that the conformations of these seven prion isolates were unaltered upon serial passage in Tg(M109) mice.

## Retrotransmission of prion strains

**Figure 4:**
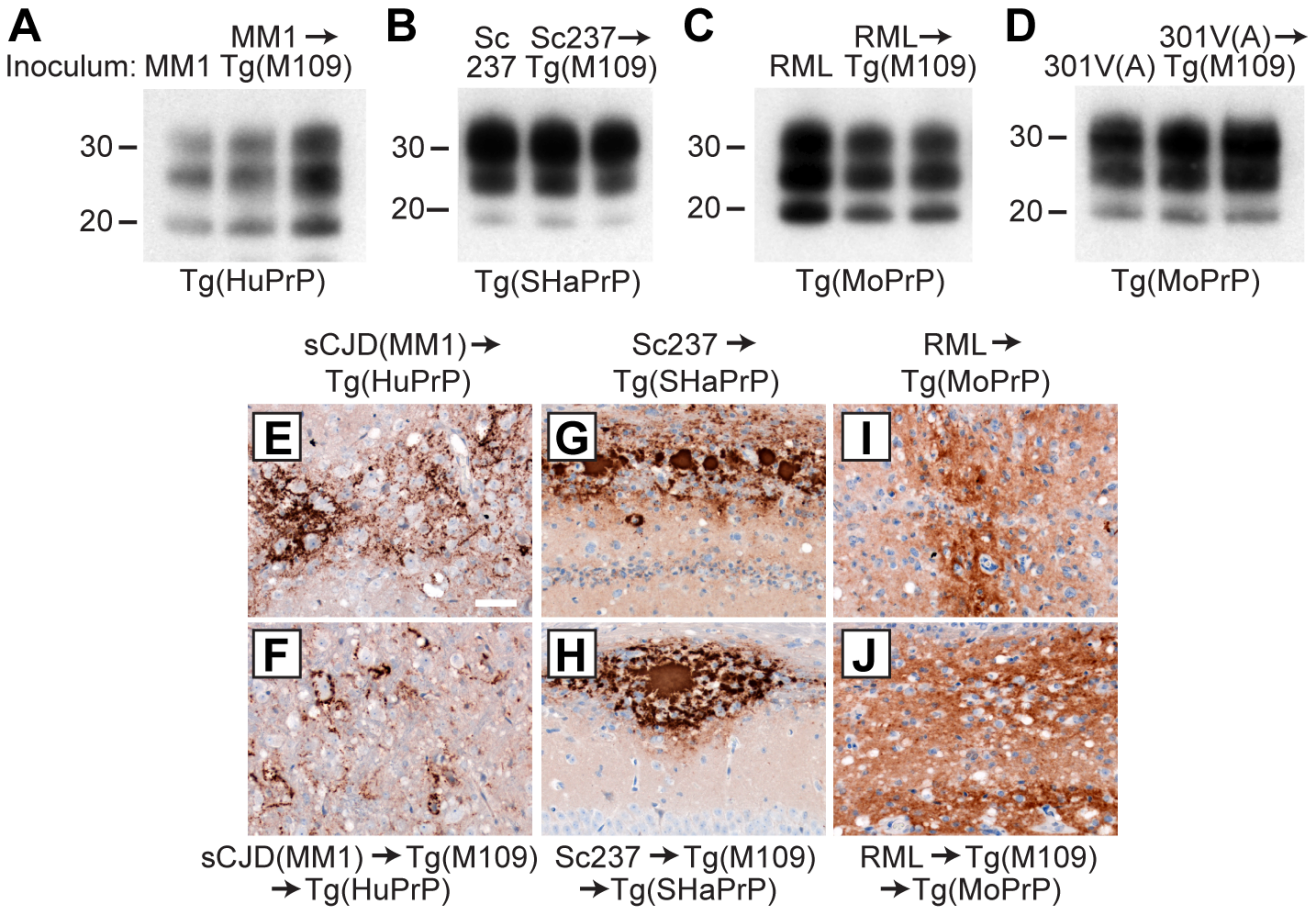
Analysis of PK-resistant PrP^Sc^ and cerebral PrP^Sc^ deposition following retrotransmission of Tg(M109)-passaged prion isolates. Following retrotransmission of Tg(M109)-passaged prion isolates, the biochemical characteristics (**A**–**D**) and deposition patterns (**E**–**J**) of PrP^Sc^ were similar. sCJD(MM1) (**A, E, F**); Sc237 (**B, G, H**); RML (**C, I, J**); and 301V(A) (**D**) prions were respectively injected into Tg(HuPrP), Tg(SHaPrP), Tg(MoPrP), and Tg(MoPrP) mice. The same inocula were also passaged once in Tg(M109) mice, then injected into the same respective lines. In the immunoblots, the electrophoretic mobility and glycosylation profile are identical in mice infected with the original inoculum and with the inoculum passaged in Tg(M109) mice. Each of the duplicate lanes for the retrotransmission samples represents an individual mouse. PK-resistant PrP^Sc^ was detected using the antibody HuM-P. In the micrographs, PrP^Sc^ was detected in the brainstem using antibody 3F4 (**E**–**F**) and in the hippocampus using antibody HuM-D18 (**G**–**J**). In panels A–D, molecular weight measurements are shown in kDa. Scale bar in E represents 50 µm and applies to panels F–J.

As a fourth test to assess the fidelity of prion strain replication upon passage in Tg(M109) mice, we performed retrotransmission experiments for the sCJD(MM1), CWD, Sc237, RML, and 301V(A) isolates. In these experiments, Tg(M109)-passaged prions were reintroduced into Tg mice expressing the PrP sequence of the species from which the prion isolate was originally derived. Inoculation of Tg(HuPrP) mice with Tg(M109)-passaged sCJD(MM1) prions, Tg(SHaPrP) mice with Tg(M109)-passaged Sc237 prions, and Tg(MoPrP) mice with Tg(M109)-passaged RML or 301V(A) prions resulted in clinical signs of prion disease in all of the inoculated animals (**Table 2**). In contrast, none of the Tg mice expressing elk PrP developed signs of neurologic illness following challenge with Tg(M109)-passaged CWD prions, suggesting that a substantial species barrier exists when attempting to convert elk PrP^C^ using bank vole PrP^Sc^. For the experiments in which successful retrotransmission was achieved, the PK-resistant PrP^Sc^ in ill recipient mice was identical to that of the original isolate passaged into the same respective Tg line, as judged by the electrophoretic mobilities and relative glycoform ratios (**Figure 4A–D**). Furthermore, the patterns of cerebral PrP^Sc^ deposition from the original isolate were recapitulated following retrotransmission (**Figure 4E–J**). Based on the conservation of biochemical, neuropathological, and conformational properties of the prion isolates upon transmission to Tg(M109) mice and upon retrotransmission after passage into Tg(M109) mice, we conclude that prion strain fidelity was often maintained upon transmission to Tg(M109) mice.

**Table 2:** Retrotransmission of Tg(BVPrP,M109)-passaged prion isolates.*

Inoculum	Recipient Line	Mean incubation period ± SEM (d)	Signs of neurologic dysfunction (*n/n*_0_)
RML → Tg(M109)	Tg(MoPrP)	169±18	8/8
301V(A) → Tg(M109)	Tg(MoPrP)	184±5	7/7
Sc237 → Tg(M109)	Tg(SHaPrP)	71±0	8/8
CWD → Tg(M109)	Tg(ElkPrP)	>523	0/6
sCJD(MM1) → Tg(M109)	Tg(HuPrP)	166±1	7/7

*n, *number of positive mice;* n*_0_, number of examined mice.*

## Transmission of prions to Tg(I109) mice

**Figure 5:**
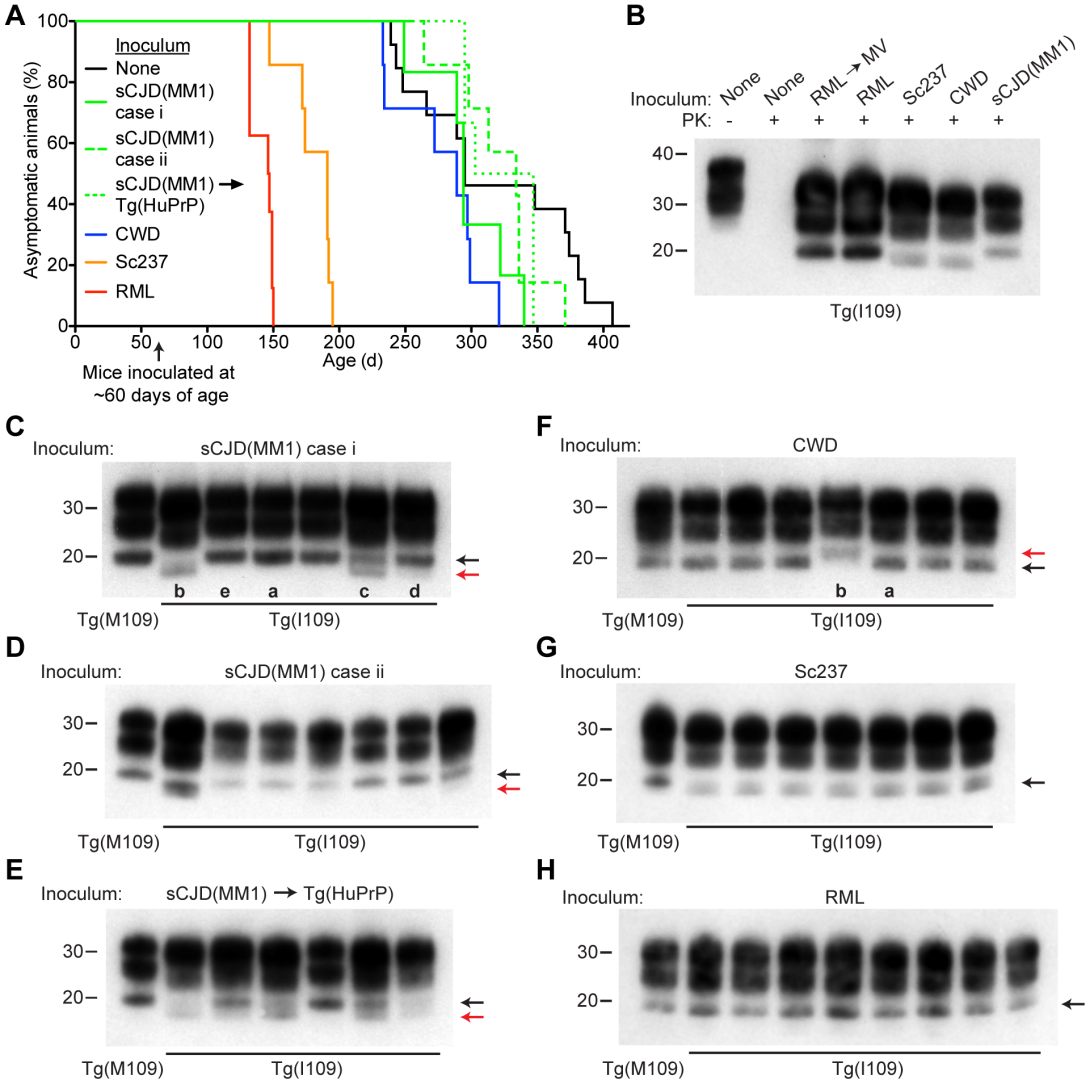
Prion strain diversity following passage of sCJD(MM1) and CWD prions in Tg(I109) mice. (**A**) Age-adjusted Kaplan-Meier survival curves for Tg(I109) mice inoculated with sCJD(MM1) prions (two independent cases) or sCJD(MM1) prions that were passaged in Tg(HuPrP) mice (green lines, *n* = 6–7 each); CWD prions (blue line, *n* = 7); Sc237 prions (orange line, *n* = 7); or RML prions (red line, *n* = 8). There was substantial overlap between the appearance of spontaneous signs of neurologic illness in uninoculated Tg(I109) mice (black line, *n* = 13) and the survival of Tg(I109) mice inoculated with CWD and sCJD(MM1) prions. For all inoculation experiments, the mice were inoculated at ∼60 days of age. (**B**–**H**) Analysis of PK-resistant PrP^Sc^ in the brains of Tg(I109) mice inoculated with sCJD(MM1) (**C**, **D**); sCJD(MM1) prions that were passaged in Tg(HuPrP) mice (**E**); CWD (**F**); Sc237 (**G**); and RML prions (**H**). With the exception of one lane in panel B (−), all samples were digested with PK. All inoculation experiments resulted in the generation of PK-resistant PrP^Sc^. Brain homogenate from an uninoculated, 171-d-old asymptomatic Tg(I109) mouse (None) is shown as a control. In panels C–H, each lane represents the PK-resistant PrP^Sc^ in the brain of an individual animal within the experiment. For each prion isolate, PK-resistant PrP^Sc^ in the brain of an infected Tg(M109) mouse is shown for comparison. Whereas inoculation of Tg(I109) mice with RML or Sc237 prions resulted in PK-resistant PrP^Sc^ conformers with the same electrophoretic mobility as those present in Tg(M109) mice (black arrows), inoculation with CWD or sCJD(MM1) isolates resulted in the emergence of novel PK-resistant PrP^Sc^ conformers with different electrophoretic mobilities (red arrows) in some animals. For all immunoblots, loading quantities were adjusted prior to immunoblotting to give similar signal intensities across all samples. Lowercase letters below blots identify samples referred to in the main text. In panels B–H, molecular weight measurements are shown in kDa. PrP was detected using the antibody HuM-P.

We inoculated Tg(BVPrP,I109)3574 mice, denoted Tg(I109), with 7 prion isolates from 5 different species: sCJD(MM1) [2 human cases and 1 case passaged in Tg(HuPrP) mice], CWD (elk), Sc237 (hamster), and RML (mouse and MV-passaged). Hemizygous Tg(I109) mice express PrP at ∼4 times the level of PrP expression found in wt mice and developed spontaneous signs of neurological dysfunction at a mean age of ∼340 days [33]. Similar to the results obtained in Tg(M109) mice, all inoculated Tg(I109) mice developed signs of progressive neurologic dysfunction (**Figure 5A**), with mean incubation periods ranging from ∼50 days for MV-passaged RML prions to ∼260 days for each of the 3 sCJD(MM1) isolates (**Table 3**). The mean incubation periods were slightly longer in Tg(I109) mice than in Tg(M109) mice on first passage of these isolates, which was likely due to the lower level of PrP expression in the Tg(I109) line. PK-resistant PrP^Sc^ (**Figure 5B**), vacuolation (**Figure S3A–E**), astrocytic gliosis (**Figure S3F–J**), and cerebral PrP^Sc^ deposition (**Figure S3K–O**) were observed in the brains of the ill Tg(I109) mice, which confirmed the diagnosis of prion disease. The ages at which the sCJD(MM1)- and CWD-inoculated Tg(I109) mice developed neurologic disease partially overlapped with the onset of spontaneous illness in this line (**Figure 5A**). However, we could distinguish the spontaneous disease phenotype from the inoculated disease because the spontaneously ill animals did not exhibit PrP 27–30 in their brains [33]. Thus, any inoculated animal that developed signs of neurologic illness but lacked detectable levels of PrP 27–30 in its brain was excluded from the study. Importantly, only four such mice were found, and the vast majority of inoculated animals (49 of 53) exhibited PrP 27–30 in their brains (**Figure 5C–H**).

**Table 3:** Transmission of diverse prion isolates to Tg(BVPrP,I109)3574 mice.*

Prion isolate	PrP^Sc^ sequence	Mean incubation period ± SEM (d)	Signs of neurologic dysfunction (*n*/*n*_0_)
RML → MV	meadow vole	49±2	8/8
RML	mouse (PrP-A)	87±2	8/8
Sc237	hamster	129±6	7/7
CWD	elk	224±13	7/7
sCJD(MM1) case i	human	243±12	6/6
sCJD(MM1) case ii	human	266±13	7/7
sCJD(MM1) → Tg(HuPrP)	human	269±11	6/6

*n, *number of positive mice;* n*_0_, number of examined mice.*

We next sought to determine whether the biochemical and neuropathological properties of the various prion isolates were conserved upon transmission to Tg(I109) mice. Tg(I109) mice inoculated with two cases of sCJD(MM1) prions or with sCJD(MM1) prions previously passaged in Tg(HuPrP) mice exhibited considerable prion strain diversity among individual animals (**Figure 5C–E**). Whereas some of the animals exhibited type 1 PrP^Sc^ similar to that observed in Tg(M109) mice (**Figure 5C**, lanes “a” and “e”), others displayed a type 2 pattern (**Figure 5C**, lane “b”) or even a mixed type 1/type 2 phenotype (**Figure 5C**, lanes “c” and “d”). Similarly, passage of CWD into Tg(I109) mice resulted in 6 of 7 animals harboring PK-resistant PrP^Sc^ similar to that observed in CWD-inoculated Tg(M109) mice (**Figure 5F**, lane “a”); one Tg(I109) mouse showed PrP^Sc^ of slower electrophoretic mobility (**Figure 5F**, lane “b”). In contrast to replication in Tg(I109) mice, neither sCJD(MM1) nor CWD prions underwent any detectable biochemical changes in PrP^Sc^ during multiplication in Tg(M109) mice (**Figure S4**). The PK-resistant PrP^Sc^ present in the brains of RML- and Sc237-inoculated Tg(I109) mice were similar to the those observed in Tg(M109) mice inoculated with the same isolates (**Figure 5G, H**), as judged by glycoform ratios and type 1 electrophoretic mobility. The patterns of cerebral PrP^Sc^ deposition in the brains of sCJD(MM1)-, Sc237-, and RML-inoculated Tg(I109) mice were similar to those observed in Tg(M109) mice inoculated with the same prion isolates (compare **Figure S3K, M, N** with **Figure 2A, M, O**). In contrast, a Tg(I109) mouse infected with CWD (also shown in **Figure 5F**, lane “a”) harbored small amounts of diffuse PrP^Sc^ in the thalamus (**Figure S3L**) whereas CWD-inoculated Tg(M109) mice had large plaque-like deposits of PrP^Sc^ (**Figure 2I**). Collectively, these results argue that passage of sCJD(MM1) and CWD prions through Tg(I109) mice resulted in alterations to these prion strains.

## Discussion

Here we demonstrate that Tg mice expressing BVPrP are highly susceptible to a diverse range of prion isolates derived from eight different species, arguing that the susceptibility of bank voles to a wide array of prions is encoded within the amino acid sequence of BVPrP itself. Although we did not challenge Tg(BVPrP) mice with every known prion isolate, we speculate that BVPrP may be a “universal acceptor” for prions. Moreover, prion strain fidelity, as judged by the molecular signatures of PK-resistant PrP^Sc^, patterns of cerebral PrP^Sc^ deposition, conformational stability, and retrotransmission experiments, was largely maintained upon transmission of many isolates to Tg(M109) mice, despite the rapid incubation periods observed upon serial passage. We note several caveats to this conclusion: (1) similarities in PrP^Sc^ molecular signatures or histopathological staining patterns do not always correlate with conservation of prion strain features [41]; (2) restoration of prion strain properties following retrotransmission has also been observed in cases where strain properties were clearly altered upon primary passage in animals [42], [43] or following extensive selection in cultured cells [44]; and (3) the dramatic reduction in incubation period observed for several isolates upon second passage in Tg(M109) mice implies that a substantial transmission barrier had been crossed, which often causes a change in strain properties [13].

The incubation periods upon primary passage for certain prion isolates, such as BSE, sCJD(MM2), sCJD(VV2), and 301V, in Tg(M109) mice were shorter than in M109 bank voles [24], [25], which is likely explained by the overexpression of BVPrP in Tg(M109) mice. In contrast, the incubation periods for sCJD(MM1) and CWD prions were similar in both Tg(M109) mice and bank voles [24], [27] whereas the incubation period for sheep scrapie prions was actually shorter in bank voles than in Tg(M109) mice [25]. Although it is difficult to make accurate comparisons between the data obtained in Tg(M109) mice and bank voles because the specific prion isolates used were different in most cases, we suggest that the generation of neurotoxic prion conformers may be limited by the PrP^C^ concentration for some prion strains (such as BSE) but not for others [such sCJD(MM1) and CWD].

### Using Tg(M109) mice to study sporadic and variant CJD prions

The study of human prions in mice has been hindered traditionally by long incubation periods. For example, sCJD(MM1) prions transmit poorly to wt mice; only a few inoculated mice ever develop prion disease and those that do exhibit incubation times of 600 days or more [12]. In Tg(HuPrP) mice, the incubation periods were ∼160 days for sCJD(MM1) prions [45] and ∼700 days for vCJD prions [18]. Reductions in the incubation times were achieved when human-specific residues in PrP were reverted to those of the mouse. For instance, in Tg mice expressing a chimeric human/mouse PrP containing 7 human residues, the incubation times for sCJD(MM1) and vCJD prions were ∼110 and ∼360 days, respectively [45]. Reversion of an additional human-specific residue to its mouse equivalent in Tg1014 mice further reduced the incubation periods to ∼80 days for sCJD(MM1) prions and ∼200 days for vCJD prions, but a change in strain type was apparent in some vCJD-inoculated animals [21]. Incubation times of ∼200 days on first passage and ∼175 days on serial passage for sCJD(MM1) prions were substantially longer in the Tg(M109) mice compared to Tg1014 mice. Notably, vCJD prions transmitted disease in ∼40 days on second passage in Tg(M109) mice, and the fidelity of the vCJD strain was maintained. To the best of our knowledge, this is the most rapid human prion strain isolated to date. The incubation periods for the MM2 and VV2 subtypes of sCJD prions upon serial passage in Tg(M109) mice were also considerably more rapid than those observed in mice expressing human PrP or chimeric human/mouse PrP [45], [46], [47]. We speculate that Tg(M109) mice inoculated with BVPrP-adapted sCJD or vCJD prions may constitute an excellent system for performing initial assessments of the *in vivo* efficacy of candidate CJD therapeutics, although weak therapeutic effects may be harder to discern in mice with such rapid incubation periods and positive results would need to be confirmed in Tg mice expressing human PrP. Based on the studies with chimeric human/mouse PrP described above, constructing chimeric human/bank vole PrP transgenes may lead to even shorter incubation times for CJD prions.

### BSE prions in Tg(M109) mice

The rapid incubation periods and apparent strain fidelity observed for most prion isolates upon serial passage in Tg(M109) mice should greatly facilitate the study of the biochemical and structural basis of prion strains. For instance, the incubation periods for BSE prions in Tg mice expressing bovine PrP is ∼250 days [19], but merely ∼60 days upon second passage in Tg(M109) mice. Thus, Tg(M109) mice may be useful for rapidly producing BSE prions for structural studies. Tg(M109) mice should also facilitate accurate comparisons between various prion strains or isolates. For instance, there has been considerable debate as to whether the conformational stability of a given prion strain is related to its incubation period. Although some of us (S. J. D. and S. B. P.) as well as others found that there was a direct correlation between conformational stability and incubation period, with less stable strains propagating more rapidly [48], [49], another study found the opposite, namely that strains with short incubation periods exhibited higher conformational stabilities [50]. We did not observe a definitive relationship between conformational stability and incubation period for seven different prion isolates serially propagated in Tg(M109) mice. One caveat of this conclusion is that we did not include any synthetic or anchorless prion strains in our study, which exhibit the highest conformational stabilities [48], [49], [51], [52].

### BVPrP and neurotoxic prion conformers

Although Tg(M109) mice developed signs of neurologic illness following challenge with a diverse range of prion isolates, for many of the strains tested, levels of PK-resistant PrP^Sc^, cerebral PrP^Sc^ deposition, and vacuolation were lower than those generally found in prion-infected rodents. Similarly low levels of PrP^Sc^ were reported in I109 bank voles inoculated with CWD prions [27]. Several explanations seem plausible: one possibility might be that BVPrP^Sc^ replicates in a few critical regions in the CNS that produce progressive neurological deficits before widespread accumulation of BVPrP^Sc^ occurs [53], [54]. A second possibility is that the amino acid sequence of BVPrP favors protease-sensitive conformations more readily than most other PrPs, similar to the predominance of protease-sensitive prions in the brains of CJD patients [55]. A third possible explanation is that during prion replication, BVPrP^Sc^ may exhibit a greater propensity for generating highly neurotoxic PrP conformers, such as the hypothetical PrP^L^ entity [22], [56], compared to PrPs from other species. The rapid production of highly neurotoxic but PK-sensitive BVPrP^Sc^ conformers may be sufficient to elicit signs of neurological deficits prior to the extensive accumulation of PK-resistant PrP^Sc^ in the brain. Indeed, Tg(I109) mice developed spontaneous signs of neurologic disease and prion-specified neuropathological changes in the absence of detectable levels of PrP 27–30 [33], suggesting that BVPrP may be inherently prone to adopting neurotoxic conformations.

### Features of a universal prion acceptor

Although BVPrP is overexpressed in the brains of Tg(BVPrP) mice, protein overexpression is insufficient to explain the general susceptibility of these mice to prions because bank voles, which express physiological levels of BVPrP, are also highly susceptible to a diverse range of prion isolates [24], [25], [27]. Therefore, an important unanswered question is what structural feature of BVPrP^C^ makes it so susceptible to forming PrP^Sc^ when exposed to PrP^Sc^ molecules from many other species? Because the mature forms of BVPrP and MoPrP differ at only eight positions [25], our results argue that at most, eight residues in PrP mediate this phenomenon. At these eight positions, six of the BVPrP residues are also found in the sequence of hamster PrP (**Figure S5**). Because hamsters do not exhibit a bank vole–like general susceptibility to prions [57], [58], it seems reasonable to speculate that the other two residues (Glu227 and Ser230) in BVPrP may play an important role in its unique behavior, especially because Glu227 is not found in other mammalian PrPs (**Figure S5**). Indeed, these two residues are located near the C-terminal end of the protein, in proximity to the GPI anchor attachment site. Although it is unclear how these residues influence the behavior of BVPrP, two possibilities include perturbation of PrP shedding from the membrane by ADAM proteases [59] and modulation of the interaction of BVPrP with other proteins or membrane lipids [32], [60], [61].

Although C-terminal residues in BVPrP may contribute to its unique properties, other BVPrP residues, either alone or in combination, may also be important. For example, unlike mouse PrP^C^, the structure of BVPrP^C^ includes a so-called “rigid loop” in the region connecting β-strand 2 to α-helix 2 [34]. Tg mice expressing either a chimeric elk/mouse PrP, a chimeric horse/mouse PrP, or the I109 variant of BVPrP, all of which contain a rigid loop, develop a spontaneous neurologic illness reminiscent of prion disease [33], [62], [63], suggesting that the presence of a rigid loop may render PrP more prone to misfolding. However, although the existence of a rigid loop in the structure of PrP^C^ can modulate the interspecies transmission of prions in some instances [64], it does not in other cases [65]. Thus, while the rigid loop in BVPrP may contribute to its unique promiscuity for diverse prion strains, it is unlikely to be the sole factor.

Although the mechanism by which BVPrP^C^ seems to act as a “universal acceptor” of prions is unknown, the structure of BVPrP^C^ might permit it to bind promiscuously to PrP^Sc^ molecules from many different species, enabling prion replication. An alternate explanation is that a misfolding intermediate on the pathway to PrP^Sc^ formation is more readily populated or is stabilized by the BVPrP sequence [66], [67]. Such a replication intermediate may be partially unfolded and thus exhibit a lower energy barrier to conversion by PrP^Sc^ from different species. This hypothesis would also explain the increased propensity for BVPrP to spontaneously adopt an infectious, neurotoxic conformation [33].

### The codon 109 polymorphism and prion strain selection

The mechanism by which some prion isolates, such as sCJD(MM1) and CWD, underwent changes upon propagation in Tg(I109) mice remains enigmatic. One possibility is that the simultaneous presence of injected prions and spontaneously formed prions in Tg(I109) mice could alter the properties of the inoculated prion isolates, because the incubation periods for CWD and sCJD(MM1) prions overlapped substantially with the occurrence of spontaneous disease in this line (**Figure 5A**). However, this explanation seems unlikely for CWD prions because their properties were also clearly altered upon serial propagation in I109 bank voles [27], which do not develop spontaneous neurologic disease. A second possibility is that the natural CWD and sCJD(MM1) isolates used in transmission experiments are not homogeneous and that less abundant conformers present in the inocula may preferentially propagate in Tg(I109) mice. Mixtures of strains have been described in both sCJD patients and in CWD-infected cervids [68], [69], [70]. In this scenario, these substrains fail to emerge as the dominant species in Tg(M109) mice or in Tg mice expressing homotypic PrP due to prion strain interference effects. Indeed, there are documented examples in which the replication of a faster but less abundant prion strain is suppressed by the presence of a slower, but more abundant strain [71], [72]. Thus, the presence of isoleucine at codon 109 of BVPrP may hinder prion interference effects, allowing less abundant but more rapid strains to gradually emerge upon serial passage. Still another hypothesis is that prion strains are actually “quasi-species” that are composed of a collection of substrains that can interconvert [73]. The energy landscape of prion replication may be very different for BVPrP(I109), allowing substrains that are not densely populated in the original host to emerge.

### Concluding remarks

The extraordinary promiscuity of BVPrP demonstrates that a small number of amino acid differences in PrP can profoundly alter the properties of prions. It is interesting to consider whether BVPrP-like versions of other aggregation-prone proteins may exist in certain species. With the recent convergence of scientific evidence that many, if not most, neurodegenerative diseases are caused by proteins that become prions [74], [75], the identification of organisms expressing Aβ, tau, or α-synuclein proteins that exhibit an increased propensity to misfold may facilitate studies on the transmissibility of Alzheimer's disease and Parkinson's disease.

## Materials and Methods

### Ethics statement

All mouse studies were carried out in accordance with the recommendations of the *Guide for the Care and Use of Laboratory Animals* (Institute of Laboratory Animal Resources, National Academies Press, Washington, DC); protocols were reviewed and approved by the UCSF Institutional Animal Care and Use Committee: “Breeding colony and production of transgenic rats and mice” (AN084871) and “Incubation periods of prion and other neurodegenerative diseases” (AN084950).

### Mouse lines

Hemizygous Tg(BVPrP,M109)22019 [“Tg(M109)”], Tg(BVPrP,M109)3118 mice, and Tg(BVPrP,I109)3574 [“Tg(I109)”] mice express BVPrP under the control of the hamster PrP promoter [33] and were maintained by backcrossing to FVB mice lacking murine PrP expression (*Prnp* ^0/0^ mice) [76]. Tg(SHaPrP)7 mice that express hamster PrP [16], Tg(ElkPrP)12584 mice expressing elk PrP [39], and Tg(HuPrP)2669 mice expressing human PrP containing the M129 polymorphism [77] were also maintained on a *Prnp* ^0/0^ background. Tg(MoPrP)4053 mice expressing the PrP-A allotype of mouse PrP [78] were maintained on a wild-type (*Prnp* ^+/+^) background.

### Prion isolates

The following prion isolates were used in this study: mouse-adapted scrapie strain RML (maintained in wild-type CD-1 mice expressing the PrP-A allotype); hamster-adapted scrapie strain Sc237; MV-passaged RML or Sc237 prions [79]; mouse-adapted BSE strain 301V (passaged in mice expressing either PrP-A or PrP-B); SSBP/1 sheep scrapie prions derived from a pool of scrapie-infected sheep brains, which were a generous gift from Dr. Nora Hunter; CWD prions derived from the brain of a naturally infected elk [Elk1 isolate; [39]]; BSE prions derived from the brain of a naturally-infected cow and then passaged 4 times in Tg mice expressing bovine PrP; human sCJD prions obtained from the brains of patients exhibiting either the MM1, MM2, or VV2 disease subtypes; sCJD(MM1) prions that were passaged in either Tg(HuPrP) mice or in guinea pigs [80]; and human prions obtained from the brain of a variant CJD patient, provided by the UK National CJD Surveillance Unit.

### Prion inoculations and mouse bioassays

Brain homogenates [10% (wt/vol) in calcium- and magnesium-free PBS] were diluted to 1% (wt/vol) using 5% bovine serum albumin (BSA). Weanling mice (∼2-month-old) were anesthetized with isoflurane and then inoculated with 30 µL of the 1% brain homogenate into the right parietal lobe using a 27-gauge syringe. Inoculated animals were assessed daily for routine health and checked three times weekly for the presence of signs of neurologic illness. Mice were euthanized once two or more neurologic signs were apparent, using the standard diagnostic criteria for assessing prion disease in mice [81]. Brains were then removed, and either snap-frozen on dry ice and then stored at −80°C for biochemical analyses or fixed in 10% buffered formalin for neuropathological studies.

### Proteinase K digestions

Ten percent (wt/vol) brain homogenates in calcium- and magnesium-free PBS were generated using either an OmniTip (Omni International) with a PowerGen homogenizer (Fisher Scientific) or with a bead beater (Precellys). Nine volumes of 10% brain homogenate were added to one volume of 10× detergent buffer [5% (vol/vol) NP-40, 5% (wt/vol) sodium deoxycholate in PBS] and then incubated on ice for 20 min followed by centrifugation at 1,000 × *g* for 5 min to remove cellular debris. Protein concentrations in the supernatant were then determined using the BCA assay (Thermo Scientific). One mg of detergent-extracted protein was diluted to a final volume of 398 µL using 1× detergent buffer [0.5% (v/v) NP-40, 0.5% (w/v) sodium deoxycholate in PBS.] Two µL of a 10 mg/mL PK stock solution (Fermentas) was then added to samples to be digested, resulting in a final PK concentration of 50 µg/mL (a PK:protein ratio of 1∶50). Samples were then incubated at 37°C with vigorous shaking for 1 h. PK digestions were terminated by the addition of phenylmethylsulfonyl fluoride (PMSF) to a final concentration of 2 mM. One hundred µL of a 10% (vol/vol) solution of sarkosyl was then added to bring the final sarkosyl concentration to 2%. Samples were then ultracentrifuged at 100,000× *g* for 1 h at 4°C, and the supernatants removed by aspiration. Pellets were resuspended in 1× NuPAGE loading buffer (Life Technologies) containing 2.5% (vol/vol) β-mercaptoethanol by vortexing, boiled for 10 min, and then analyzed by immunoblotting.

### Immunoblotting

PK-digested brain homogenate samples (containing 200–500 µg of digested total protein) were prepared as described above and then loaded onto 10% NuPAGE gels (Life Technologies). Undigested samples (typically 10 µg total protein) were prepared by diluting detergent-extracted brain homogenate directly into 1× NuPAGE loading buffer containing β-mercaptoethanol and then boiling for 5 min. SDS-PAGE was performed using the MES buffer system, and gels were subsequently transferred to PVDF membranes using a wet blotting system. Membranes were blocked for 2 h at room temperature using blocking buffer [5% (w/v) nonfat milk in Tris-buffered saline containing 0.05% (v/v) Tween-20 (TBST)] and then incubated with horseradish peroxidase (HRP)-conjugated primary antibody overnight at 4°C. Blots were washed three times with TBST, developed using the enhanced chemiluminescent detection system (GE Healthcare) and then exposed to x-ray film. PrP was detected using the antibody HuM-P [82].

### Conformational stability assays

Twenty µL of detergent-extracted brain homogenate was mixed with 2× stocks of GdnHCl to give final concentrations of 1, 1.5, 2, 2.5, 3, 3.5, or 4 M GdnHCl. For the 4.5- and 5-M samples, only 10 µL of brain homogenate was used. Samples were incubated at 22°C with shaking (800 rpm) for 2 h and then diluted to 0.4 M GdnHCl in 1× detergent buffer. PK was added to a final concentration of 20 µg/mL, and the samples were digested at 37°C with shaking for 1 h. Digestions were then terminated by adding PMSF to a final concentration of 2 mM. One hundred µL of a 12% (vol/vol) sarkosyl solution was then added to give a final concentration of 2%. Samples were then ultracentrifuged at 100,000× *g* for 1 h at 4°C, and the supernatants removed by gentle aspiration. Pellets were resuspended in 1× NuPAGE loading buffer containing β-mercaptoethanol, boiled for 10 min, and then analyzed by immunoblotting as described above. Films were scanned using a CCD camera (FluorChem 880; Alpha Innotech) and then densitometry performed using Image J. GdnHCl_1/2_ values were calculated using the variable slope (four parameter) function in Prism 5.

### Neuropathology

Brains were removed, immersion-fixed in 10% buffered formalin, and then embedded in paraffin. Sections were cut at 8 µm, mounted on glass slides, deparaffinized, and then processed for immunohistochemistry or stained with hematoxylin and eosin (H&E). Endogenous tissue peroxidases were inhibited by incubating the slides in a 3% hydrogen peroxide solution (prepared in methanol) for 30 min. Sections to be stained with anti-PrP antibodies were subjected to hydrolytic autoclaving (121°C for 10 min in citrate buffer). Slides were then blocked with 10% (vol/vol) normal goat serum for 1 h and then incubated with primary antibody overnight at 4°C. The following primary antibodies were used: anti-GFAP rabbit polyclonal antibody Z0334 (Dako, 1∶500 dilution) to detect astrocytic gliosis, and anti-PrP antibodies 3F4 (1∶1,000 dilution) [83] or HuM-D18 (1∶500 dilution) [84] to detect PrP^Sc^ deposition. Bound antibody was detected using a Vectastain ABC peroxidase kit (Vector Laboratories) and visualized using 3-3′-diaminobenzidine (DAB). Slides were counterstained with hematoxylin and then photographed using an AxioImager.A1 microscope (Carl Zeiss).

## Supporting Information

Figure S1 **Vacuolation and astrocytic gliosis in the brains of Tg(M109) mice inoculated with diverse prion isolates.** Cerebral vacuolation (H&E staining, **A**–**I**) and astrocytic gliosis (GFAP immunostaining, **J**–**R**) following inoculation of Tg(M109) mice with sCJD(MM1) (**A**, **J**); sCJD(VV2) (**B**, **K**); vCJD (**C**, **L**); BSE (**D**, **M**); CWD (**E**, **N**); scrapie SSBP/1 (**F**, **O**); Sc237 (**G**, **P**); RML (**H**, **Q**); or 301V(A) (**I**, **R**) prions. The hippocampus is shown in panels C, H, L, P, and Q; the brainstem in panels A, I, J, N, and R; the thalamus in panels D–G, K, M, and O; and the cortex in panel B. Scale bar in A represents 50 µm and applies to all panels.
(TIF)

Figure S2 **Inoculation of Tg(M109) mice with RML or Sc237 prions leads to low levels of PK-resistant PrP^Sc^.** Low levels of PK-resistant PrP^Sc^ were observed in Tg(M109) mice inoculated with RML prions (1^st^ or 2^nd^ passage) compared to levels in RML-inoculated wild-type FVB or Tg(MoPrP) mice (**A**) or with Sc237 prions (1^st^ or 2^nd^ passage) compared to levels in Sc237-inoculated hamsters (Ha) or Tg(SHaPrP) mice (**B**). In contrast, comparable amounts of PK-resistant PrP^Sc^ were observed in Tg(M109) mice inoculated with BSE or vCJD prions (2^nd^ passage) and in RML-inoculated wild-type mice (**C**). Equal amounts of PK-digested total protein were loaded in each lane. Molecular weight measurements are shown in kDa. PrP was detected using the antibody HuM-P.
(TIF)

Figure S3 **Vacuolation, astrocytic gliosis, and PrP^Sc^ deposition in the brains of Tg(I109) mice inoculated with diverse prion isolates.** Cerebral vacuolation (H&E staining, **A–E**); astrocytic gliosis (GFAP immunostaining, **F–J**); and PrP^Sc^ deposition (PrP immunostaining, **K–O**) following inoculation of Tg(I109) mice with sCJD(MM1) [**A**, **F**, **K**; isolate “e” from Figure 5C is shown]; CWD [**B**, **G**, **L**; isolate “a” from Figure 5F is shown]; Sc237 (**C**, **H**, **M**); RML (**D**, **I**, **N**); or MV-passaged RML (**E**, **J**, **O**) prions. The brainstem is shown in panels A, C, F, H, and K; the hippocampus in panels D, E, I, J, M, N, and O; and the thalamus in panels B, G, and L. PrP^Sc^ deposition was detected using the antibody HuM-D18. Scale bar in A represents 50 µm and applies to all panels.
(TIF)

Figure S4 **Absence of prion strain diversity in Tg(M109) mice inoculated with various prion isolates.** Analysis of PK-resistant PrP^Sc^ in the brains of Tg(M109) mice inoculated with sCJD(MM1) prions (two cases: **A**, **B**); sCJD(MM1) prions that were passaged in Tg(HuPrP) mice (**C**); or CWD prions (**D**). Each lane shows the PK-resistant PrP^Sc^ in the brain of an individual animal within the experiment. Unlike in Tg(I109) mice, no prion strain diversity was observed following inoculation of Tg(M109) mice with the sCJD(MM1) or CWD isolates. Prior to immunoblotting, loading quantities were adjusted to give similar signal intensities across all samples. Molecular weight measurements are shown in kDa. PrP was detected using the antibody HuM-P.
(TIF)

Figure S5 **Amino acid sequence alignment of the processed region of BVPrP with other mammalian PrPs.** Within the mature, processed region of BVPrP (residues 23–231), mouse PrP and BVPrP differ at 8 positions (boxed residues). Of these 8 residues in BVPrP, 6 are also present in the sequence of hamster PrP (red boxes) whereas Glu227 and Ser230 (green boxes) are not. Glu227 is unique to BVPrP whereas Ser230 is also present in human PrP. The location of BVPrP polymorphic residue 109, where either methionine or isoleucine is encoded, is also shown. The location of the three α-helices and the two short β-strands in the structure of BVPrP^C^ [34] are shown as blue and gray lines, respectively. Sequence alignment was performed using ClustalW2 (http://www.ebi.ac.uk/Tools/msa/clustalw2/).
(TIF)

Table S1 **Transmission of diverse prion isolates to Tg(BVPrP,M109)3118 mice.**
(DOCX)

Table S2 **Inoculation of Tg(MoPrP)4053 mice with diverse prion isolates.**
(DOCX)
