# Supplementary figures and images for: Evidence That Bank Vole PrP Is a Universal Acceptor for Prions
Source: PLoS Pathog. 2014 Apr 3;10(4):e1003990. doi: 10.1371/journal.ppat.1003990 (PMC3974871; doi:10.1371/journal.ppat.1003990)

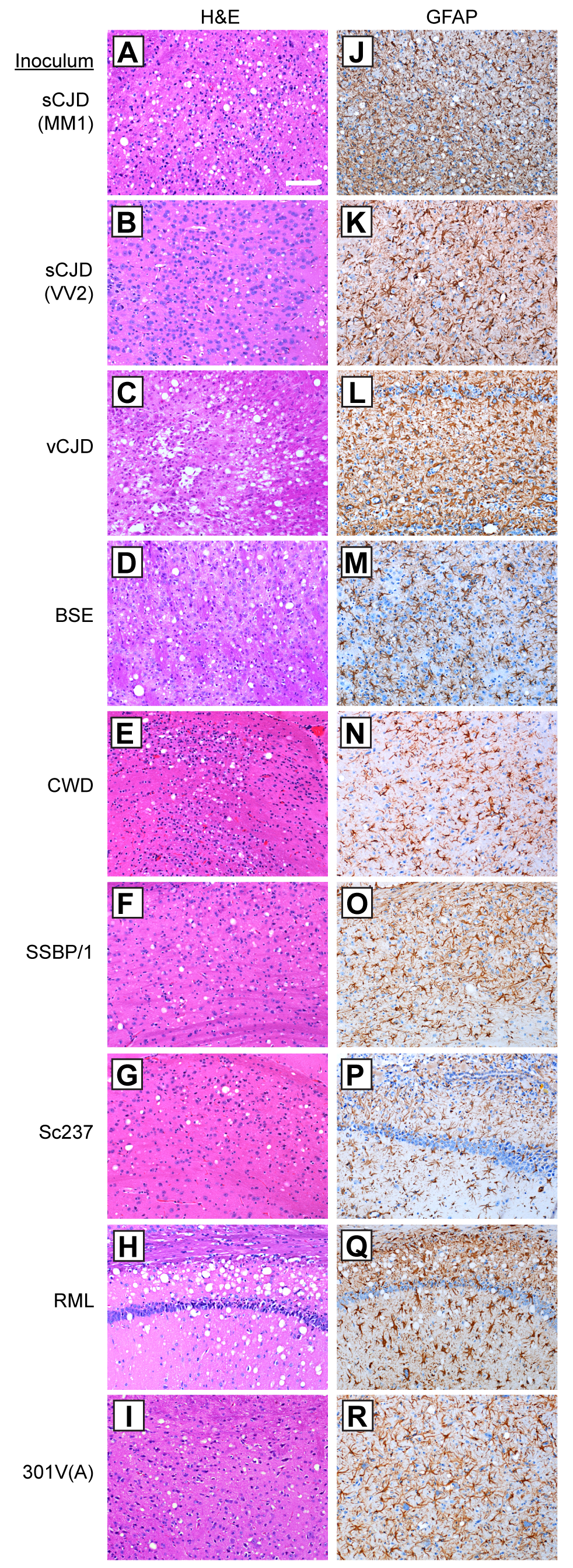

Supplement: Figure S1 — Vacuolation and astrocytic gliosis in the brains of Tg(M109) mice inoculated with diverse prion isolates. Cerebral vacuolation (H&E staining, A–I) and astrocytic gliosis (GFAP immunostaining, J–R) following inoculation of Tg(M109) mice with sCJD(MM1) (A, J); sCJD(VV2) (B, K); vCJD (C, L); BSE (D, M); CWD (E, N); scrapie SSBP/1 (F, O); Sc237 (G, P); RML (H, Q); or 301V(A) (I, R) prions. The hippocampus is shown in panels C, H, L, P, and Q; the brainstem in panels A, I, J, N, and R; the thalamus in panels D–G, K, M, and O; and the cortex in panel B. Scale bar in A represents 50 µm and applies to all panels. (TIF) [file ppat.1003990.s001.tif]

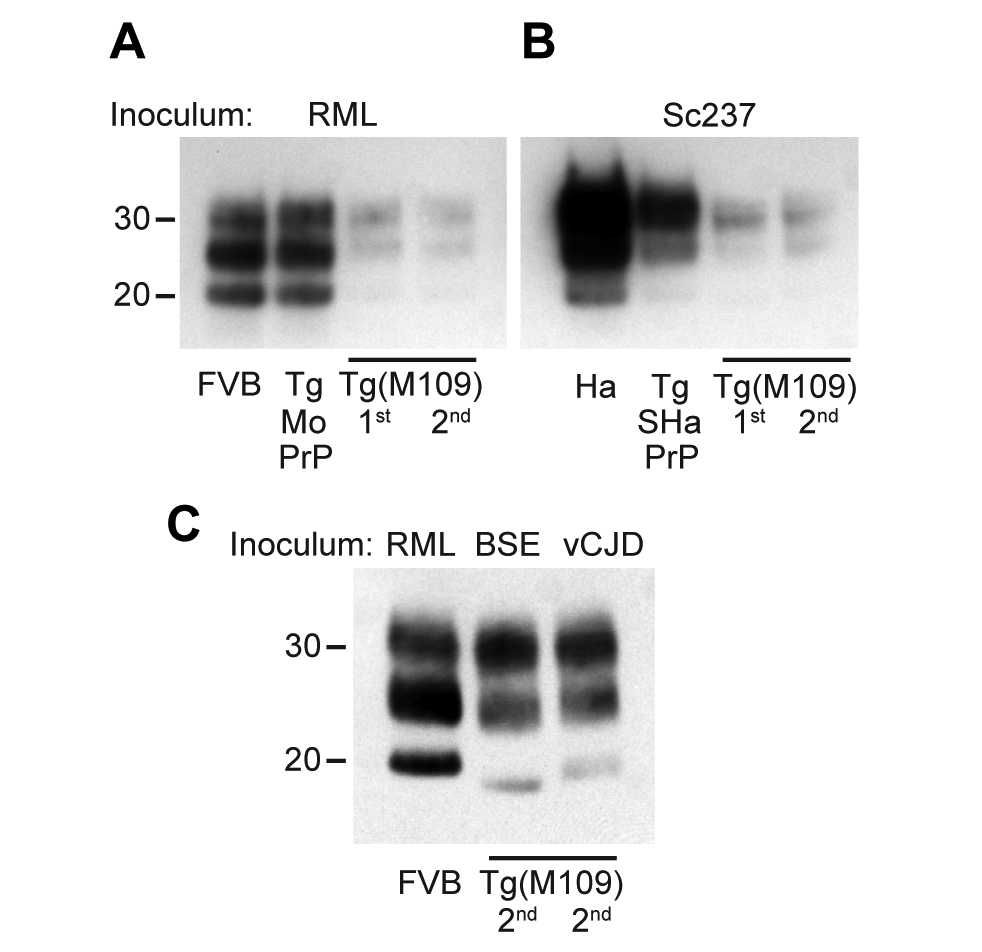

Supplement: Figure S2 — Inoculation of Tg(M109) mice with RML or Sc237 prions leads to low levels of PK-resistant PrPSc. Low levels of PK-resistant PrPSc were observed in Tg(M109) mice inoculated with RML prions (1st or 2nd passage) compared to levels in RML-inoculated wild-type FVB or Tg(MoPrP) mice (A) or with Sc237 prions (1st or 2nd passage) compared to levels in Sc237-inoculated hamsters (Ha) or Tg(SHaPrP) mice (B). In contrast, comparable amounts of PK-resistant PrPSc were observed in Tg(M109) mice inoculated with BSE or vCJD prions (2nd passage) and in RML-inoculated wild-type mice (C). Equal amounts of PK-digested total protein were loaded in each lane. Molecular weight measurements are shown in kDa. PrP was detected using the antibody HuM-P. (TIF) [file ppat.1003990.s002.tif]

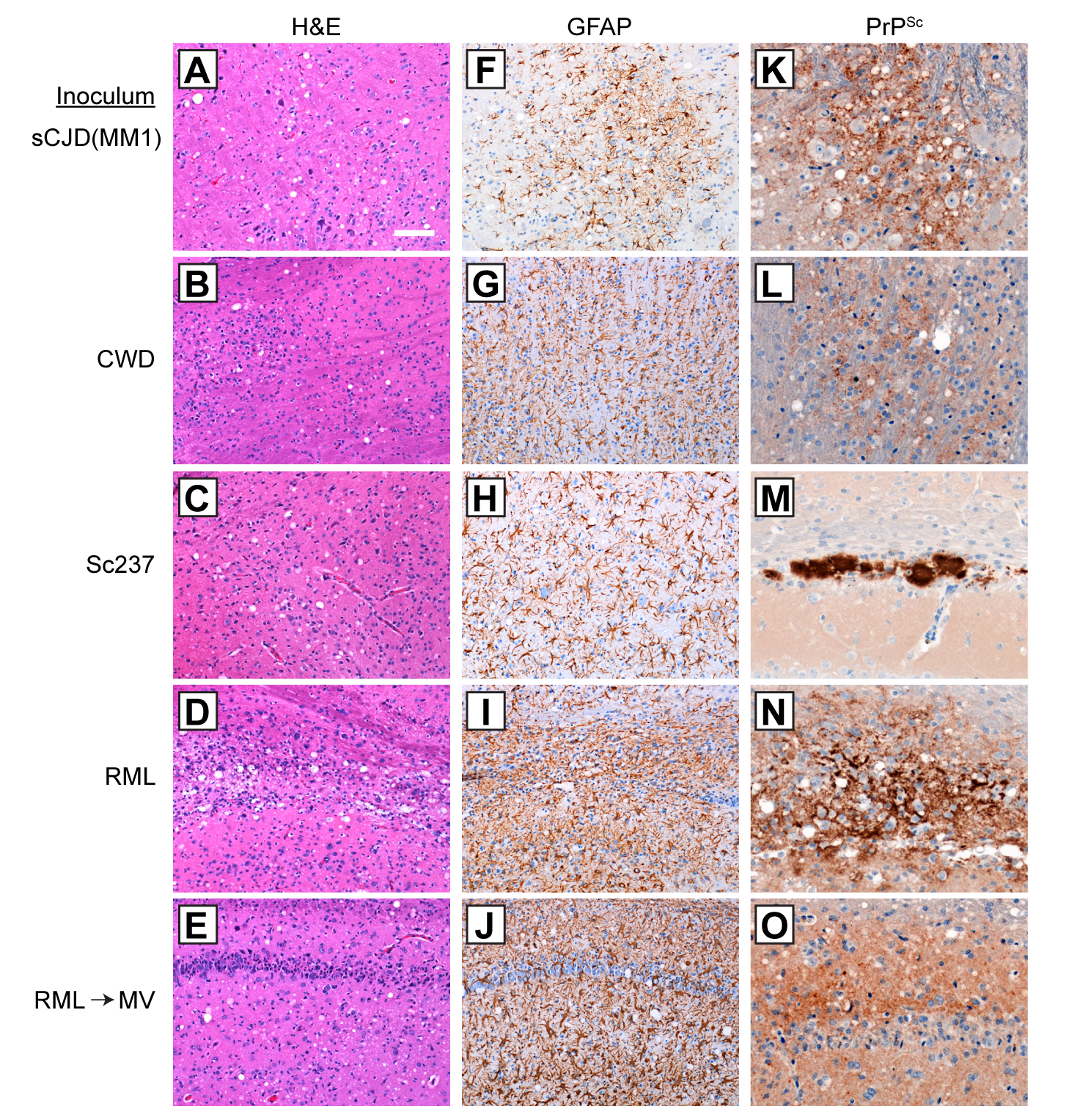

Supplement: Figure S3 — Vacuolation, astrocytic gliosis, and PrPSc deposition in the brains of Tg(I109) mice inoculated with diverse prion isolates. Cerebral vacuolation (H&E staining, A–E); astrocytic gliosis (GFAP immunostaining, F–J); and PrPSc deposition (PrP immunostaining, K–O) following inoculation of Tg(I109) mice with sCJD(MM1) [A, F, K; isolate “e” from Figure 5C is shown]; CWD [B, G, L; isolate “a” from Figure 5F is shown]; Sc237 (C, H, M); RML (D, I, N); or MV-passaged RML (E, J, O) prions. The brainstem is shown in panels A, C, F, H, and K; the hippocampus in panels D, E, I, J, M, N, and O; and the thalamus in panels B, G, and L. PrPSc deposition was detected using the antibody HuM-D18. Scale bar in A represents 50 µm and applies to all panels. (TIF) [file ppat.1003990.s003.tif]

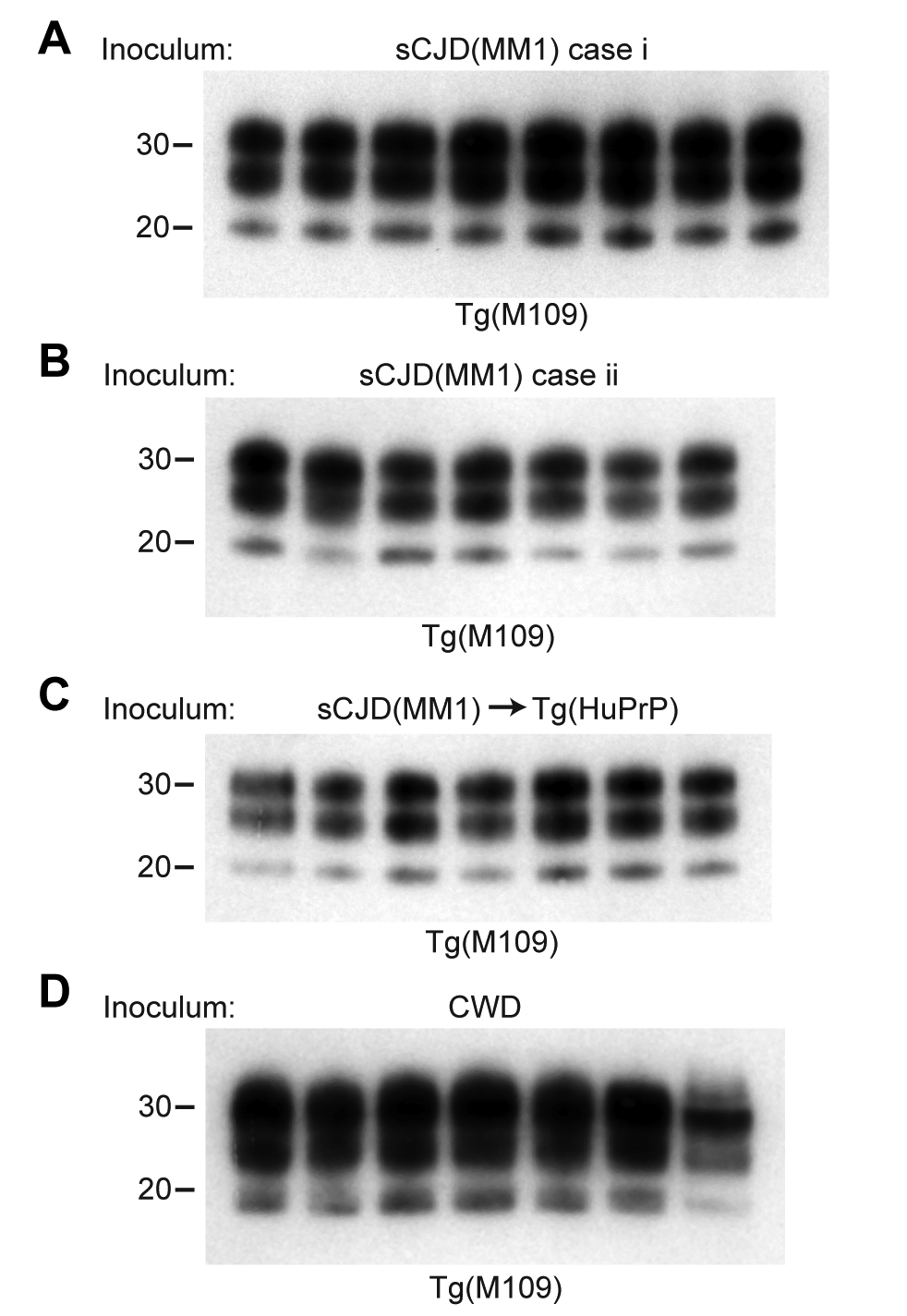

Supplement: Figure S4 — Absence of prion strain diversity in Tg(M109) mice inoculated with various prion isolates. Analysis of PK-resistant PrPSc in the brains of Tg(M109) mice inoculated with sCJD(MM1) prions (two cases: A, B); sCJD(MM1) prions that were passaged in Tg(HuPrP) mice (C); or CWD prions (D). Each lane shows the PK-resistant PrPSc in the brain of an individual animal within the experiment. Unlike in Tg(I109) mice, no prion strain diversity was observed following inoculation of Tg(M109) mice with the sCJD(MM1) or CWD isolates. Prior to immunoblotting, loading quantities were adjusted to give similar signal intensities across all samples. Molecular weight measurements are shown in kDa. PrP was detected using the antibody HuM-P. (TIF) [file ppat.1003990.s004.tif]

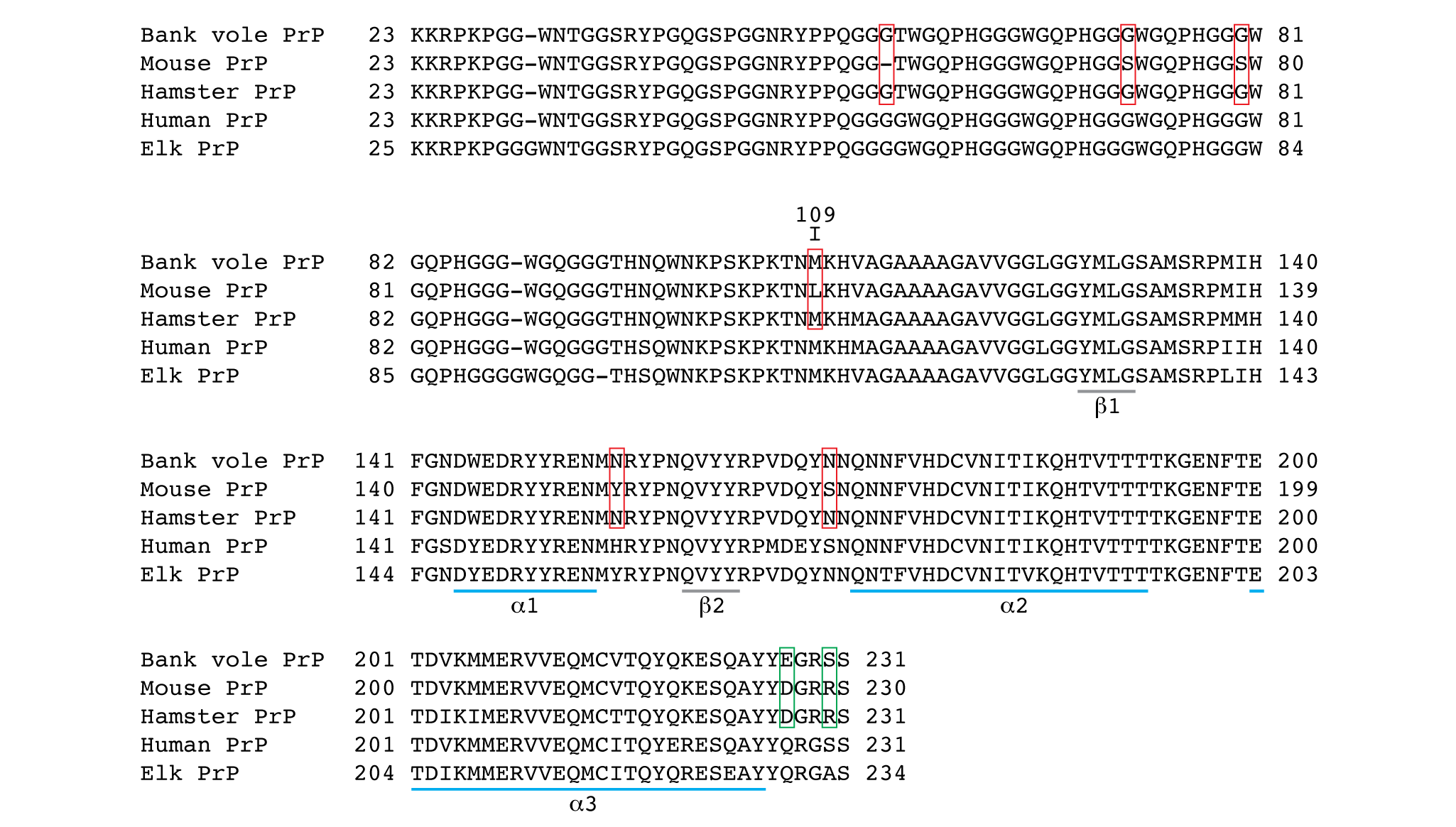

Supplement: Figure S5 — Amino acid sequence alignment of the processed region of BVPrP with other mammalian PrPs. Within the mature, processed region of BVPrP (residues 23–231), mouse PrP and BVPrP differ at 8 positions (boxed residues). Of these 8 residues in BVPrP, 6 are also present in the sequence of hamster PrP (red boxes) whereas Glu227 and Ser230 (green boxes) are not. Glu227 is unique to BVPrP whereas Ser230 is also present in human PrP. The location of BVPrP polymorphic residue 109, where either methionine or isoleucine is encoded, is also shown. The location of the three α-helices and the two short β-strands in the structure of BVPrPC [34] are shown as blue and gray lines, respectively. Sequence alignment was performed using ClustalW2 (http://www.ebi.ac.uk/Tools/msa/clustalw2/). (TIF) [file ppat.1003990.s005.tif]
