# Supplementary material for: Evidence That Bank Vole PrP Is a Universal Acceptor for Prions
Source: PLoS Pathog. 2014 Apr 3;10(4):e1003990. doi: 10.1371/journal.ppat.1003990 (PMC3974871; doi:10.1371/journal.ppat.1003990)
Supplement: Table S1 — Transmission of diverse prion isolates to Tg(BVPrP,M109)3118 mice. (DOCX) [file ppat.1003990.s006.docx]

Table S1. Transmission of diverse prion isolates to Tg(BVPrP,M109)3118 mice.*

| Prion isolate | PrP^Sc^ sequence | Mean incubation  period ± SEM (d) | Signs of neurologic  dysfunction (*n*/*n*_0_) |
| --- | --- | --- | --- |
| RML 🡪 MV | meadow vole | 60 ± 0 | 8/8 |
| RML | mouse (PrP-A) | 91 ± 5 | 7/7 |
| Sc237 🡪 MV | meadow vole | 93 ± 3 | 8/8 |
| Sc237 | hamster | 153 ± 3 | 8/8 |
| sCJD MM1 case i | human | 206 ± 6 | 7/7 |

* n, number of positive mice; n_0_, number of examined mice.
