# Supplementary material for: Evidence That Bank Vole PrP Is a Universal Acceptor for Prions
Source: PLoS Pathog. 2014 Apr 3;10(4):e1003990. doi: 10.1371/journal.ppat.1003990 (PMC3974871; doi:10.1371/journal.ppat.1003990)
Supplement: Table S2 — Inoculation of Tg(MoPrP)4053 mice with diverse prion isolates. (DOCX) [file ppat.1003990.s007.docx]

Table S2. Inoculation of Tg(MoPrP)4053 mice with diverse prion isolates.*

| Prion isolate | PrP^Sc^ sequence | Mean incubation  period ± SEM (d) | Signs of neurologic  dysfunction (*n*/*n*_0_) |
| --- | --- | --- | --- |
| RML | mouse (PrP-A) | 51 ± 2 | 12/12 |
| Sc237 | hamster | 501 | 1/7 |
| CWD | elk | 110 ± 6 | 4/8 |
| sCJD MM1 case i^†^ | chimeric mouse/human | 571 ± 0 | 2/8 |
| sCJD MM1 🡪 GP | guinea pig | 516 | 1/7 |

* n, number of positive mice; n_0_, number of examined mice.

† Isolate was passaged once in Tg1014 mice, which express chimeric mouse/human PrP.
